# Vaccination coverage in the context of the emerging Yellow Fever threat in French Guiana

**DOI:** 10.1371/journal.pntd.0007661

**Published:** 2019-08-19

**Authors:** Claude Flamand, Sarah Bailly, Camille Fritzell, Sandrine Fernandes Pellerin, Alhassane Toure, Naïssa Chateau, Mona Saout, Sébastien Linares, Fabien Dubois, Laurent Filleul, Mirdad Kazanji

**Affiliations:** 1 Epidemiology Unit, Institut Pasteur in French Guiana, Cayenne, French Guiana; 2 Clinical Coordination of Translational Research Center, Institut Pasteur, Paris, France; 3 Geographic Information and Knowledge Dissemination Unit, Direction de l’Environnement, de l’Aménagement et du logement Guyane, Cayenne, French Guiana; 4 French Public Health Agency, Paris, France; Lowell General Hospital, UNITED STATES

## Abstract

**Background:**

French Guiana, a French overseas department located in South America between Brazil and Surinam, is the only European territory geographically located in the Amazonian forest complex and is considered endemic for yellow fever (YF).

In the context of the emergent threat of YF in Latin America, we conducted a large household cross-sectional survey from June to October 2017 to estimate vaccination coverage in the population and to determine associations with sociodemographic and geographical characteristics.

**Methodology/Principal findings:**

In total, 1,415 households and 2,697 individuals were included from the 22 municipalities of French Guiana. YF vaccination coverage was estimated at 95.0% (95% CI: 93.4–96.2) in the entire territory but was spatially heterogeneous, with the lowest levels estimated in the western part of the territory along the Surinamese cross-border region, particularly in children under 16 years who were not enrolled in school, immigrant adults and disadvantaged populations with low socioeconomic indexes.

**Conclusions/Significance:**

Despite the good vaccination coverage against YF in the general population of French Guiana resulting from the compulsory nature of YF vaccination for residents and travelers, there is an urgent need to improve vaccination coverage in vulnerable populations living in the northwestern part of the territory to limit the risk of transmission in the context of the emerging YF threat in South America.

Despite the relative rarity of YF and the significant number of infectious and tropical diseases in French Guiana, clinicians should adopt a high index of suspicion for YF, particularly in vulnerable and at-risk populations.

## Author summary

Yellow fever (YF) is the most severe arbovirus to circulate in the Americas. French Guiana, a French overseas department located in South America between Brazil and Surinam, is the only European territory geographically located in the Amazonian forest complex and is considered endemic for YF. We conducted a large general population survey from June to October 2017 to estimate vaccination coverage in the population and to identify target vulnerable populations for catch-up vaccination strategies. In total, 1,415 households and 2,697 individuals were included from the 22 municipalities of French Guiana. YF vaccination coverage was estimated at 95.0% (95% CI: 93.4–96.2) in the entire territory but was spatially heterogeneous, with the lowest levels estimated in the western part of the territory along the Surinamese cross-border region, particularly in children under 16 years who were not enrolled in school, immigrant adults and disadvantaged groups of populations with low socioeconomic indexes. Our findings showed that vaccination campaigns should be prioritized and adapted to improve vaccination coverage among vulnerable populations living in the northwestern part of the territory to limit the risk of transmission in the context of the emerging YF threat in South America. Despite the relative rarity of YF and the significant number of infectious and tropical diseases in French Guiana, clinicians should adopt a high index of suspicion for YF, particularly in vulnerable and at-risk populations.

## Introduction

Yellow fever (YF) is the most severe arbovirus to circulate in the Americas, with symptoms ranging from mild non-specific illness to hemorrhagic fever, a systemic illness characterized by high viremia, hepatic, renal and myocardial injury, hemorrhage, and high lethality [1]. A single-dose vaccine has existed since the 1940s and has helped to substantially control and reduce YF transmission [2–4]. However, complete eradication is prevented by the sylvatic cycle of the virus within nonhuman primary hosts, and *Aedes aegypti* mosquitoes are responsible for occasional transmission to people [5]. Recent important outbreaks of YF in Africa and South America have confirmed the potential of arthropod-borne viruses to emerge or reemerge in risk areas [6] and have highlighted the urgent need to assess vaccination coverage efforts in the most exposed countries.

Since November 2016, after decades of silence, Brazilian authorities and scientists have reported an outbreak of YF associated with an exponential increase in the number of confirmed cases and deaths in humans [7–9]. The YF virus has spread into the coastal Atlantic forest zones and moved rapidly into the southeast and south of the country in less than one year, reaching several populous Brazilian states whose residents had not been included in the YF vaccination program [9,10]. The majority of reported cases have occurred in rural areas, clearly reflecting a typical sylvatic transmission cycle occurring between forest mosquitoes and forest-dwelling nonhuman primates, with humans serving only as accidental hosts. This important alert has prompted the Brazilian Ministry of Health to conduct massive vaccination campaigns among unvaccinated residents of affected areas [11].

French Guiana, a French overseas department located in South America between Brazil and Surinam, is the only European territory geographically located in the Amazonian forest complex and is considered endemic for YF [12]. Since 1967, YF vaccination has been compulsory in French Guiana for all individuals older than 1 year of age (with a booster dose every 10 years). The vaccination is free of charge and widely accessible in public vaccination centers and by accredited private practitioners. In February 2016, according to the Strategic Advisory Group of Experts on Immunization and the modifications of the International Health Regulations [13], French health authorities adopted the use of only a single dose of vaccine for most residents and travelers.

Over the last decades, *Ae*. *aegypti* has been responsible for several major dengue fever outbreaks [14–18] and for the recent emergence of chikungunya in late 2013 [19–21] and Zika in 2016 [22,23]. Considering the large number of travelers moving through the Brazilian river border, the recent outbreak of YF has raised particular concern that an urban transmission could occur in French Guiana, specifically for nonvaccinated population subgroups, through the *Ae*. *aegypti* mosquitoes that are strongly represented in the territory.

Although vaccination coverage reported by recent assessments was better than that observed 20 years ago, some geographical areas may present unsatisfactory levels of coverage, particularly in forest and exposed environments [24–27]. While a survey conducted in 2000 estimated YF vaccine coverage of 80–90% in children under 15 years old [24], the overall vaccine coverage was estimated at 95.9% (95% CI 95·5–96·3) in 2009 in 9339 children from primary and secondary schools [25]. Lower coverage rates between 75% and 81% were observed in small municipalities located outside the urbanized and coastal areas.

Moreover, French Guiana is experiencing continuous major waves of immigration facilitated by the natural and uncontrollable quality of the river borders, particularly from countries where vaccination against YF is not mandatory.

In this context, two sporadic and fatal cases of YF were reported in French Guiana 1 year apart [28], confirming that sylvatic YF circulation is active in this territory, particularly among nonvaccinated populations involved in important activities in the forest environment. The first case was confirmed in August 2017 in a 43-year-old Brazilian woman with unknown vaccination status. Epidemiological investigations reported a history of stay in the forest, suggesting that the patient could have been contaminated either in the Amapá state in Brazil or in French Guiana. In August 2018, a second case was biologically confirmed in a non-vaccinated Swiss-citizen 47-year-old man who had entered through a river border and had lived in French Guiana for 4 months. Epidemiological investigations reported regular work activities on forest roads, suggesting autochthonous transmission in the forest environment.

The last autochthonous case was identified in 1998 in the southeast of the territory[29]. These two recent case reports illustrate that despite the compulsory nature of YF vaccination in this French overseas region since 1967, maintaining focus on the need for YF vaccination is important, especially in areas with favorable ecosystems for YF transmission.

In the context of the emergent threat of YF in Latin America and, consequently, in French Guiana, we conducted a general population cross-sectional study to estimate vaccination coverage in the population and to determine associations with sociodemographic and geographical characteristics.

## Methods

### Ethics statement

The study was approved by the “Sud-Ouest & Outre-Mer IV” Ethical Research Committee (No. CPP17-007a/2017-A00514-49) and by the French Data Protection Authority (No. DR-2017-324), which is responsible for ethical issues and the protection of individual data collection.

#### Study design and population

**Fig 1:**
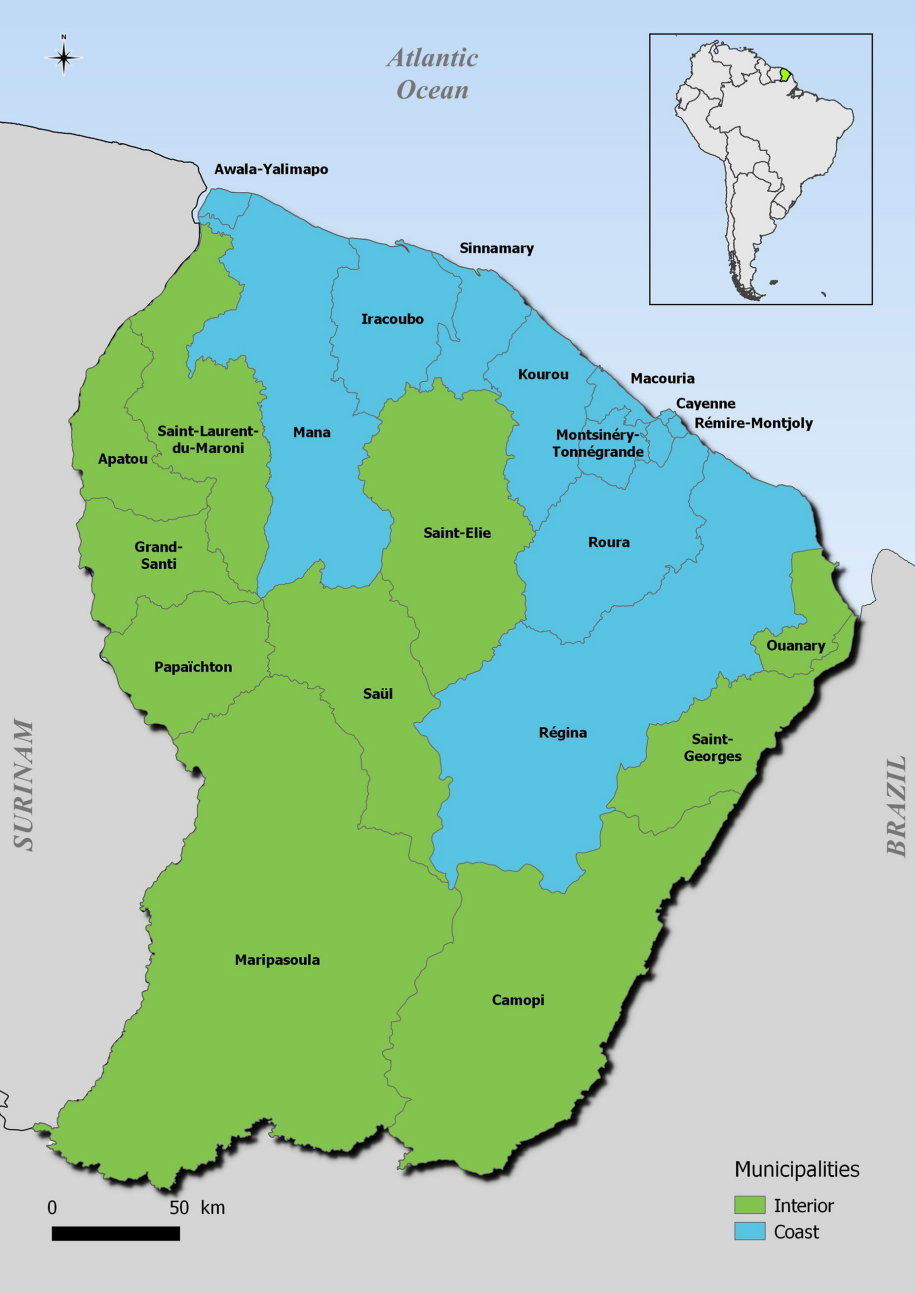
Map of French Guiana, South America, geodata source: OpenStreetMap; QGIS 2.18 software.

We conducted a cross-sectional vaccination coverage study through a household-based survey from June to October 2017 involving residents of the 22 municipalities of French Guiana. In 2017, 259,865 inhabitants lived in French Guiana, primarily in two main geographical regions: an urbanized coastal strip area along the Atlantic Ocean named the “coastal area”, where a large part of the population lives, and a more remote area along the Surinamese and Brazilian frontiers named “interior” (Fig 1).

To reach the desired sample size (2,500 individuals), a total of 1,600 households were randomly selected for possible participation in the study from household databases maintained by the Geographic Information and Knowledge Dissemination Unit of the Regional Agency for Environment, Planning and Housing and the National Institute of Economic and Statistical Information (INSEE) [30].

### Procedures

Publicity and information about the survey were provided through the media and contact with local and national authorities. Fieldworker teams were trained to visit all households, explain the project objectives, and, when allowed, collect participants’ signatures on a free and informed consent form and conduct the interviews. All household members of selected households who were 2–75 years of age were invited to take part in the study. For all participants under 18 years of age, one or two responsible adults signed the informed consent form. Data were collected through a standardized questionnaire installed on tablets to register demographic, socioeconomic and household characteristics. Vaccination cards or any other proof-of-vaccination documents were requested from all participants. Vaccination status was based on presented vaccination cards and verbal reports of vaccination when vaccination cards were not available.

### Statistical analysis

We employ the following notations to describe the study design:

- *i*: one of the 22 strata (municipalities);
- *M*_*i*:_ number of primary sampling units (households) in the *i*^th^ stratum, *i =* 1, …, 22;
- *S*_*i*:_ number of primary sampling units (households) selected from the *i*^th^ stratum, *i =* 1, …, 22;
- *mi*: number of primary sampling units (households) actually enrolled in the study from the *i*^th^ stratum, i = 1, …, 22;
- *P*_*i*_: number of individuals living within the *i*^th^ stratum, *i =* 1, …, 22 (census data);
- *p*_*i*_: number of individuals actually enrolled in the study from the *i*^th^ stratum, *i =* 1, …, 22.

We considered that in each municipality *i*, the probability of selecting a particular subject was equal to the probability of selecting the subject’s household and was (*m*_*i*_*/M*_*i*_), corresponding to a statistical weight equal to (1/ *m*_*i*_*/M*_*i*_*)*_*=*_ (*M*_*i*_*/m*_*i*_*)*. This statistical weight indicates the number of people in the population represented by each subject in the sample.

We applied a post-stratification adjustment to each of these weights to arrive at the final statistical weight for each subject. This adjustment helped us to weight the age-sex groups within each municipality to match the distribution in the total population of French Guiana. Ten age groups ([2–5 years [, [5–10[, [10–15[, [15–20[, [20–25[, [25–35[, [35–45[, [45–55[, [55–65[, and ≥65 years) were defined within male and female groups. For each age-sex subgroup, we applied an adjustment factor *c*_*ijk*_ to obtain a final statistical weight:

*w*_*ijk*_ *= (M*_*i*_*/m*_*i*_)*c_*ijk*,_ where *i* indexes municipalities, j indexes sex groups and k indexes age groups.

We constructed a household socioeconomic index combining a multiple correspondence analysis and a hierarchical cluster analysis based on household material possessions, socioprofessional category and household income.

The weighted vaccination coverage estimation for one dose of YF was based on doses recorded on vaccination documents and/or reported by participants.

Associated factors were identified using survey-weighted Poisson regression, and the strength of selected variables and vaccination coverage was estimated by raw and adjusted risk ratios (RR) and a 95% confidence interval (CI). All RRs excluding 1.0 were considered significant. Analyses were conducted using the survey capabilities of Stata version 15 statistical software (Stata Corp, College Station, TC, USA)[31]. French Guiana’s layers were drawn using geodata from OpenStreetMaps (http://www.openstreetmap.org), and mapping operations were performed using QGIS 2.18 software [32].

## Results

In total, 1,415 households and 2,697 individuals were included from 22 municipalities (Table 1), representing 58% of eligible household members. The mean household size was 1.9 individuals [range: 1 to 11]. The mean age was 30.5, ranging from 2 to 75 years old. Comparison of the sociodemographic characteristics of the study sample with census data demonstrated an overrepresentation of women (58.9% vs. 50.0% in the general population of French Guiana) and adults over 25 years (64% vs. 53% in French Guiana). These differences were accounted for in the analyses of vaccination coverage and risk factors by allocating a poststratification weight to each participant. Vaccination coverage for YF (at least one dose) was estimated at 95.0% (95% CI: 93.2–96.3) throughout the entire region of French Guiana (Table 1). Eighty percent of the respondents presented a vaccination certificate or an equivalent document as evidence of vaccination, while 15.6% reported that they had received the vaccination but had no hard evidence. The number of booster doses received by vaccinated individuals ranged from 1 to 6. The mean age at vaccination was 1.7 years among children under 10 born in French Guiana.

**Table 1:** Vaccination coverage by municipalities and sub-municipalities, EPIARBO study, French Guiana.

Geographical areas	Population size	Number of individuals enrolled	*Vaccination certificate*	*Weighted Vaccination coverage*
*i*	*P*_*i*_	*p*_*i*_	% [95% CI]	% [95% CI]
Cayenne	57,614	446	87.9 [83.8–91.1]	98.5 [96.3–99.4]
Matoury	32,427	266	91.3 [86.8–97.6]	99.6 [97.4–99.9]
Saint-Laurent	43,600	301	49.4 [41.1–57.8]	76.9 [67.7–84.1]
Kourou	26,221	293	91.4 [86.5–94.6]	98.8 [96.5–99.6]
Remire-Montjoly	23,976	192	83.1 [76.7–88.1]	99.5 [96.6–99.9]
Macouria	11,719	164	87.8 [80.0–92.8]	98.9 [96.1–99.7]
Mana	10,241	96	65.3 [53.9–75.1]	92.7 [84.2–96.8]
Maripasoula municipality	11,856	145	76.6 [68.1–83.4]	97.9 [91.6–99.5]
Maripasoula center area	.	77	66.7 [53.1–77.9]	96.1 [85.4–99.0]
Twenke-Talhuen village	.	33	87.7 [76.2–94.1]	100
Antecume-Pata village	.	35	89.3 [75.7–95.7]	100
Apatou	8,431	62	61.8 [47.5–74.3]	92.1 [78.9–97.3]
Grand-Santi	6,969	61	32.7 [18.3–51.2]	62.3 [39.3–80.9]
Saint-Georges	4,020	86	69.5 [56.8–79.9]	91.3 [82.1–96.0]
Papaïchton	7,266	49	33.4 [21.2–48.4]	78.3 [60.2–89.6]
Sinnamary	2,957	39	85.3 [68.8–93.8]	100
Roura municipality	3,713	70	92.5 [81.6–97.2]	98.8 [91.8–99.8]
Roura main area	.	45	100	100
Cacao village	.	25	79.5 [58.1–91.5]	96.6 [80.5–99.5]
Montsinnery-Tonnegrande	2,473	66	92.5 [80.9–97.3]	99.1 [93.5–99.9]
Iracoubo	1,878	53	78.5 [65.2–87.7]	100
Regina	946	75	75.4 [62.1–85.1]	98.3 [89.7–99.8]
Regina center area	.	64	75.3 [60.3–85.9]	98.1 [88.2–99.7]
Kaw village	.	11	76.0 [46.2–92.1]	100
Camopi	1,769	115	67.5 [55.5–77.6]	95.9 [89.9–98.4]
Camopi center area	.	83	63.2 [48.3–75.9]	99.0 [93.0–99.9]
Trois-Sauts village	.	32	80.6 [78.4–83.6]	88.3 [69.4–96.2]
Awala	1,379	60	83.8 [67.2–92.9]	94.8 [85.1–98.3]
Saint-Elie	95	11	56.1 [27.2–81.4]	82.4 [47.4–96.0]
Ouanary	165	13	70.2 [58.4–79.8]	100
Saül	150	34	81.3 [61.5–92.2]	100
Total	259,865	2,697	80.6 [78.4–82.6]	95.0 [93.4–96.2]

**Fig 2:**
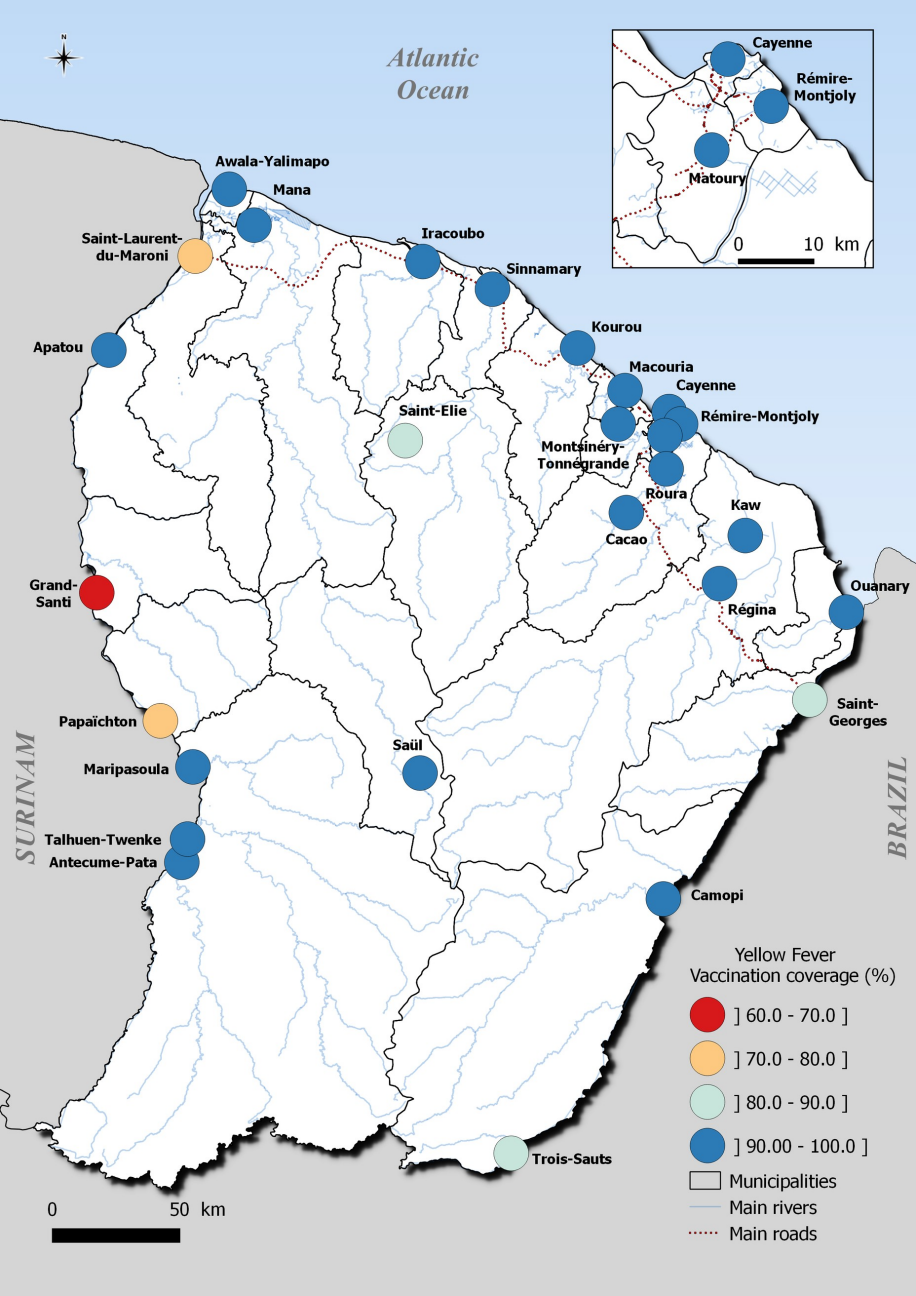
Spatial distribution of vaccination coverage in French Guiana, EPIARBO study, geodata source: OpenStreetMap; QGIS 2.18 software.

The coverage was spatially heterogeneous and decreased from the central coastal area to the western part of the territory along the Maroni River, which forms the border with Suriname (Fig 2). The highest vaccination coverage levels were observed in small and remote villages or in municipalities with fewer than 3,000 inhabitants (Antecume Pata, Twenke-Talhuen, Iracoubo, Roura, and Sinnamary), where all the respondents were vaccinated.

While the majority of French Guiana had coverage levels higher than 90%, three municipalities located in the west border area (Fig 2) had relatively low levels of vaccination coverage, including Grand-Santi (62.3%, 95% CI: 39.3%–80.9%), Saint Laurent (76.9%, 95%CI: 67.7%-84.1%) and Papaïchton (78.3%, 95% CI: 60.2%-89.6%).

More importantly, vaccination coverage was particularly low in children under 16 years in the municipalities of Saint-Laurent (40.1%, 95% CI: 21.6–61.9), Papaïchton (51.9%, 95% CI: 26.0–76.8) and Grand-Santi (52.7%, 95% CI: 25.1–78.7), while the coverage level was estimated at 97.1%, 95% CI: 94.5–98.5 in the other municipalities. More than 40% (N = 23/51) of unvaccinated children aged 3–16 years living in these municipalities were not enrolled in school. Most of them (91%) had parents born in Surinam or Guyana, and 72% of the children were born in French Guiana. Nearly 70% of the unvaccinated adults living in these municipalities were born outside of the country, and 30% had lived in French Guiana for less than 10 years. Disadvantaged groups benefiting from universal health coverage and state medical assistance schemes (specifically conceived for undocumented migrants who become eligible after 3 months of residency in the French territory) and those with low socioeconomic indexes were also associated with lower vaccination coverage in the entire territory (Table 2).

**Table 2:** Factors associated with vaccination coverage, EPIARBO study, French Guiana.

Characteristic	Total enrolled individuals	Weighted coverage (%) [95% CI]	Pearson test p-value	Raw RR [95% CI]	Adjusted RR [95% CI]
**Gender**			0.23		
Male	1,108	95.6 [93.8 96.8]		Ref	-
Female	1,589	94.4 [92.2–96.1]		0.98 [0.97–1.01]	
**Age, years**			<10^−3^		
[2–5[	62	80.5 [67.2–89.7]		Ref	Ref
[5–15[	494	90.6 [86.2–93.7]		1.10 [0.82–1.48]	1.12 [0.99–1.26]
[15–25[	413	95.7 [927.-97.5]		1.17 [0.87–1.58]	1.14 [1.01–1.28]
[25–35[	471	96.6 [94.4–97.9]		1.19 [0.89–1.59]	1.14 [1.01–1.28]
[35–45[	442	98.1 [96.4–99.0]		1.20 [0.90–1.61]	1.15 [1.02–1.29]
[45–55[	362	98.3 [96.3–99.2]		1.21 [0.90–1.63]	1.15 [1.02–1.30]
[55–65[	284	99.2 [97.4–99.7]		1.23 [0.91–1.66]	1.16 [1.03–1.30]
≥ 65	169	98.8 [96.4–99.6]		1.21 [0.88–1.66]	1.14 [1.02–1.29]
**Birth place**			<10^−3^		
French Guiana	1,481	93.0 [90.4–94.9]		Ref	Ref
Surinam	213	88.3 [81.7–92.7]		0.95 [0.89–1.01]	0.95 [0.89–1.01]
Brazil	174	98.5 [95.7–99.5]		1.06 [1.03–1.09]	1.03 [1.01–1.05]
Other South America	63	92.3 [73.9–98.1]		0.99 [0.89–1.11]	1.05 [1.01–1.10]
Haiti	223	97.7 [94.4–99.1]		1.05 [1.01–1.08]	1.05 [1.01–1.08]
Caribbean Island	136	99.7 [97.6–99.9]		1.07 [1.05–1.10]	1.02 [1.01–1.03]
Europe	349	100		1.08 [1.05–1.10]	1.01 [1.01–1.03]
Asia	28	100		1.08 [1.05–1.10]	1.01 [0.99–1.03]
Africa	22	100		1.08 [1.05–1.10]	1.01 [0.99–1.02]
Others	8	100		1.08 [1.05–1.10]	1.04 [0.99–1.10]
**Health insurance status**			<10^−3^		
General/professional social coverage	1,330	98.9 [97.6–99.5]		Ref	Ref
Universal Health Coverage (CMU)	1,233	90.8 [87.3–93.4]		0.92 [0.89–0.95]	0.97 [0.94–0.99]
State Medical Assistance (AME)	128	90.6 [83.9–94.6]		0.92 [0.86–0.97]	0.93 [0.88–0.99]
No health care	6	71.9 [34.4–92.9]		0.72 [0.46–1.15]	0.49 [0.47–1.16]
**Household size**			0.02		
[1–2]	536	98.8 [97.3–99.5]		Ref	-
[3–6]	1,359	95.5 [93.1–97.1]		0.97 [0.94–0.99]	
≥ 6	592	92.9 [87.8–95.9]		0.94 [0.90–1.98]	
**Household income**			<10^−3^		
<1,000 €	565	94.9 [91.6–96.9]		Ref	-
[1,000€-3,000€ [	833	96.9 [94.0–98.4]		1.02 [0.98–1.06]	
[3,000€-5,000€ [	285	98.9 [92.8–99.8]		1.04 [1.01–1.08]	
≥ 5,000€	153	99.4 [95.9–99.9]		1.05 [1.01–1.08]	
Not documented	861	93.5 [88.5–96.4]		0.94 [0.89–0.99]	
**Socioeconomic index**			<10^−3^		
Low	233	88.4 [80.9–93.2]		Ref	Ref
Intermediate	908	94.2 [90.8–96.4]		1.07 [0.99–1.14]	1.05 [0.97–1.14]
Elevated	1,291	98.5 [97.2–99.5]		1.11 [1.03–1.19]	1.00 [1.01–1.15]
School enrollment*			<10^−3^		
Yes	531	92.2 [87.5–95.2]		Ref	
No	46	50.5 [30.3–70.5]		0.55 [0.36–0.84]	-

*Children aged 3–16 years

## Discussion

In 2017, epizootic and sporadic human cases were observed in the northern part of the state of Pará, Brazil, near French Guiana. A recent case in neighboring Suriname was also identified in the Brokopondo Lake area, less than 100 km from the river border with French Guiana [28,33]. These situations suggest ongoing viral circulation and an emerging threat in the wider Guiana Shield region. In this context, it was incumbent upon us to estimate YF vaccination coverage throughout the entire region of French Guiana.

Our results highlight good vaccination coverage against YF in the general population of French Guiana resulting from the compulsory nature of YF vaccination for residents and travelers [34].

However, vaccination rates appear to be insufficient in some western cross-border areas connected by river routes (outside of vaccination control) to countries potentially lacking sufficient vaccination coverage. This includes areas or countries where recent epidemics have occurred or where vaccination against YF is not mandatory [9,33].

While vaccination coverage estimates in French Guiana were the lowest in some western cross-border areas, including Grand Santi (62.3%), Papaïchton (78.3%), and Saint-Laurent (76.9%), the actual vaccination rate in these municipalities may be lower than the level recommended by the WHO to achieve and maintain a protective population-level immunity (assumed to be approximately 60–80%) [35]. This concern is based on the very low proportion of individuals who provided proof of vaccination in this part of French Guiana.

Importantly, a large number of unvaccinated individuals in these areas were out-of-school children who were consequently not involved in vaccination monitoring and catch-up strategies conducted in formal educational settings. This situation poses an additional challenge for health authorities and prevention operators to reach and include these populations in vaccination catch-up strategies. While we estimated the population of children aged 3–16 years not attending school in the entire territory to be 5.4%, 75% of them lived in these western cross-border municipalities.

Furthermore, it is possible that our study tends to overestimate vaccination coverage, particularly in specific areas associated with a low proportion of presentation of vaccination proof. Although a large majority of individuals who reported that they had received vaccination without hard evidence were able to provide the date and the service of vaccination, information on vaccination history without a card or hard evidence could be falsified.

Another limitation of our study is that irregular immigrants without health coverage were underrepresented in our sample. Given that individuals without health coverage could not be enrolled in our survey because of restrictions from French legislation, this population was underrepresented in our study. Although this population was very small in most of the main municipalities of the coastal areas, some households were excluded in the western part of the territory, which is known for high levels of immigration, because the adults and referents of the selected household did not have health insurance status.

Six individuals without health insurance status were included from households whose referents were eligible and enrolled in the survey. Only three of them had received single doses of vaccination, suggesting that recent immigrants, who are often in irregular situations and targeted by police operations, are at risk of being unvaccinated and are difficult for health professionals to reach. This situation may lead to YF cases or clusters in specific and unvaccinated population subgroups.

In this context, it should be a priority to focus vaccination campaigns in the northwestern part of the territory where vaccination coverage rates are the lowest and most likely overestimated. Vaccination strategies and campaigns should be adapted to continuously improve vaccination coverage in children who are not enrolled in school, migrant populations who have recently arrived in French Guiana and other vulnerable populations, particularly if they are involved in essential activities in forest areas. Although the Social Security Fund and local health authorities provide yellow fever vaccination free of charge in the entire territory, vulnerable and unvaccinated populations without health insurance status may have poor access to health care providers and limited opportunities to be considered in the vaccination campaigns that are usually conducted in school and health centers. Alternative community initiatives based on places of religious worship, citizen’s centres or other places for social gathering should be used to reach these populations to increase vaccination coverage in target populations. Moreover, it is essential to maintain vaccine strategies and policies related to airport vaccination status controls and to raise awareness among health-care providers regarding the importance of verifying the immunization status of patients at each encounter regardless of patient origin to contribute to vaccination catch-up efforts.

Despite the relative rarity of YF and the significant number of infectious and tropical diseases in French Guiana, clinicians should adopt a high index of suspicion for YF, particularly in unvaccinated travelers returning from affected regions.

Daily air travel exchanges with metropolitan France could be the basis for the introduction of the YF virus in Europe. Despite the low epidemic risk in temperate countries, the local cycle of YF transmissions in regions where competent *Aedes albopictus* populations are established becomes a plausible scenario [36]. The latest YF case, confirmed in a Swiss man who supposedly arrived in French Guiana by land in April 2018, illustrates this hypothesis. If the clinical symptoms of the patient had not developed while he was still in French Guiana, this could have led to an imported case in Europe and consequently to the occurrence of secondary cases, underlining the need for continued vigilance with respect to YF.
